# Venenastthrombose als frühe Manifestation von COVID-19

**DOI:** 10.1007/s00347-021-01492-4

**Published:** 2021-09-15

**Authors:** Kristin Hösel, Mark Saeger, Johann B. Roider

**Affiliations:** Universitätsklinikum Schleswig-Holstein - Campus Kiel, Arnold-Heller-Str. 3, 24104 Kiel, Deutschland

## Anamnese

Ein 55-jähriger Patient stellte sich mit seit circa 3 Tagen bestehender schmerzloser Visusminderung auf dem rechten Auge vor.

Zum Zeitpunkt der Diagnosestellung bestanden keine Grunderkrankung und keine Medikation.

## Befund

**Figure 1:**
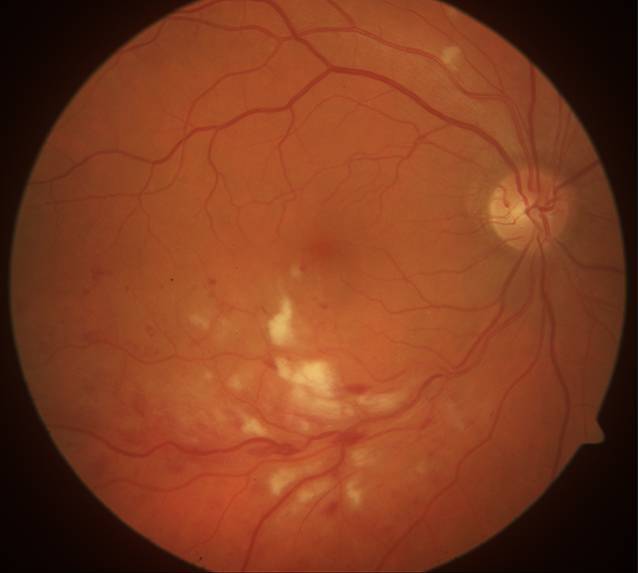

**Figure 2:**
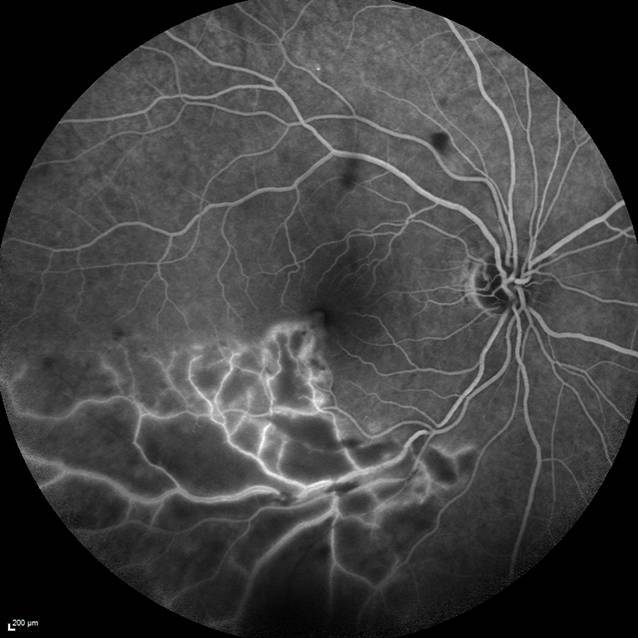

Der bestkorrigierte Visus des betroffenen rechten Auges betrug 0,5. Der Augeninnendruck sowie vordere Augenabschnitt waren unauffällig. Fundoskopisch zeigten sich dilatierte Venen des temporal-inferioren Gefäßbogens mit umgebenden Streifenblutungen sowie multiplen Cotton-Wool-Herden (Abb. 1). Es bestand keine Glaskörperinfiltration. In der optischen Kohärenztomographie (OCT) zeigte sich ein zystoides Makulaödem. In der Fluoreszeinangiographie zeigte sich eine Venenastthrombose (VAT) des inferioren Gefäßbogens mit umgebender retinaler Ischämie sowie Schrankenstörung (Abb. 2).

Der Visus des Partnerauges betrug 0,8. Inferior der Papille des linken Auges zeigte sich eine winzige retinale Hämorrhagie unterhalb der Papille bei sonst unauffälligem ophthalmologischem Status.

## Diagnose

Der Patient wurde mit der Diagnose einer Venenastthrombose (Auge rechts) stationär aufgenommen.

## Therapie und Verlauf

Am Tag der stationären Aufnahme wurde ein Nasen-Rachen-Abstrich auf SARS-CoV-2-RNA untersucht (RT-PCR), welcher sich positiv zeigte. Auch auf Nachfrage verneinte der Patient Allgemeinsymptome, insbesondere Luftnot, Husten oder Geschmacksstörungen. Die Infektionskette war nicht bekannt. Die Körpertemperatur betrug 35,6 °C, der Blutdruck 151/98 mm Hg, die Herzfrequenz 82/min und die Sauerstoffsättigung 98 %. Die gängigen Entzündungsmarker waren im Normbereich (CRP = 2,5 mg/l, LDH = 177 U/l, Troponin T = 5,4 ng/l).

Wir führten einen Bindehautabstrich (Schirmer-Streifentest) auf beiden Augen durch. In beiden Augen konnte SARS-CoV-2-RNA nachgewiesen werden.

Der Patient wurde auf unsere Isolierstation verlegt und erhielt 100 mg Prednisolon intravenös über 5 Tage mit folgender Entlassung in die häusliche Quarantäne.

Am fünften Tag nach Entlassung (etwa 14 Tage nach Auftreten der Sehstörung) wurde der Patient erneut stationär in der Klinik für Innere Medizin aufgenommen. Er berichtete nun über zunehmende Luftnot, Verschlechterung des Allgemeinbefindens, Fieber und Husten. Die Körpertemperatur betrug 38,4 °C, der Blutdruck 124/84 mm Hg, die Herzfrequenz 91/min und die Sauerstoffsättigung 92 %. Auch die Entzündungsparameter zeigten einen Anstieg: CRP = 71,3 mg/l, LDH 286 U/l, Troponin T = 6,3 ng/l. Eine Computertomographieaufnahme zeigte das Bild einer Pneumonie in moderater Ausprägung, suggestiv für eine SARS-CoV-2-Pneumonie und ohne Nachweis einer Lungenarterienembolie. Die ophthalmologische Untersuchung erbrachte den gleichen Befund wie 1 Woche zuvor. Der Bindehautabstrich war jedoch zu dem Zeitpunkt auf beiden Augen negativ für SARS-CoV-2-RNA.

Während des stationären Aufenthaltes verbesserte sich sein Befund, und er konnte nach 7 Tagen erneut mit einem guten Allgemeinbefinden entlassen werden.

Der Patient erhielt eine Anschlussbehandlung mit intravitrealen Antivascular-growth-factor-Injektionen ins rechte Auge (insgesamt bisher 4 Injektionen zum Zeitpunkt der Einreichung) sowie fokale Laserkoagulation der ischämischen Areale. Hierunter kam es zu einem Rückgang des zystoiden Makulaödems im OCT mit Visusanstieg auf 0,9.

## Diskussion

Der Patient in unserem Fall berichtete ca. 10 bis 14 Tage vor dem Auftreten von generellen COVID-19-Symptomen über Sehstörungen auf dem rechten Auge. Klinisch zeigten sich Zeichen einer Venenastthrombose (VAT) mit ausgeprägten Cotton-Wool-Spots, intraretinalen Streifenblutungen sowie Makulaödem. Insgesamt erinnert das pizzaähnliche Fundusbild auch an eine Zytomegalievirusretinitis. In der Fluoreszeinangiographie zeigte sich jedoch das Bild eines ischämischen Venenastverschlusses der inferioren Temporalvene. Auch die Minderperfusion nach einer Gefäßkreuzungsstelle spricht eindeutig für einen Venenastverschluss. Eine virusassoziierte Retinitis oder retinale Nekrose ist nicht zu sehen. Wir können nicht mit Sicherheit unterscheiden, ob die VAT durch die COVID-19-Erkrankung ausgelöst wurde oder im Zusammenhang mit systemischen Komorbiditäten zu sehen ist. Es zeigte sich neben der VAT inferior auch ein Cotton-Wool-Spot am superioren Gefäßbogen (Abb. 1). Dies ist für eine reine VAT eher untypisch und spricht für das Vorliegen gefäßassoziierter Komorbiditäten. Jedoch fanden sich in der kardiovaskulären Ursachenabklärung keine Risikofaktoren für das Auftreten eines thrombogenen Ereignisses. Ebenso gab es auf dem Partnerauge fundoskopisch bis auf eine einzelne Hämorrhagie unterhalb der Papille keine Zeichen eines chronischen arteriellen Hypertonus. Ein durchgeführtes Thrombophiliescreening blieb negativ. Wir vermuten daher eher eine Assoziation mit der COVID-19-Infektion, auch in Anbetracht der häufig berichteten vaskulären Nebenwirkungen bei COVID-19. Auch wenn der Pathomechanismus noch nicht vollständig geklärt ist, erhöhen hyperinflammatorische Reaktionen das Risiko für venöse thrombembolische Ereignisse [1]. In der SERPICO-Studie konnten retinale Gefäßaffektionen bei COVID-19-Patienten nachgewiesen werden. Die Autoren fanden heraus, dass der venöse Gefäßdurchmesser bei Erkrankten signifikant größer war als bei gesunden Probanden. Des Weiteren schien der Gefäßdurchmesser positiv mit der Schwere der COVID-19-Infektion zu korrelieren [2]. Vaskuläre Komplikationen sind auch nach COVID-19-Impfungen beschrieben. Der impfstoffassoziierten Thrombozytopenie („vaccine-induced immune thrombotic thrombocytopenia“ [VITT]) liegt wahrscheinlich eine Autoimmunreaktion auf COVID-19-Impfstoffe zugrunde, welche zu Thrombozytopenien und Thrombosen führen kann. Der genaue Pathomechanismus dieser seltenen Komplikation ist bisher unbekannt, ebenso wie die Bedeutung im Hinblick auf mögliche retinale Gefäßverschlüsse nach der Impfung [3, 4].

Interessanterweise zeigte sich außerdem an beiden Augen ein positiver Bindehautbefund auf SARS-CoV‑2. Sowohl eine Ausbreitung per continuitatem als auch hämatogen oder neurogen in die Retina sind denkbar [5]. Auch konnte bereits SARS-CoV‑2 in retinalem Gewebe (post mortem) nachgewiesen werden [6]. Retinale Manifestationen von COVID-19 sind folglich denkbar.

Unserer Erkenntnis nach, ist unser Fall der erste, welcher einen retinalen Venenastverschluss als Erstmanifestation einer COVID-19-Pneumonie beschreibt. Eine kürzlich publizierte Fallbeschreibung berichtete über einen 40-jährigen COVID-19-Patienten mit Pneumonie, tiefer Beinvenenthrombose sowie bilateralem Zentralvenenverschluss [7]. Auch gibt es eine Reihe weiterer Case Reports über retinale Verschlüsse bei jungen, gesunden COVID-19-Patienten [8, 9]. Berichte über retinale Thrombosen nach COVID-19-Impfungen sind nach unserem Wissen jedoch bisher nicht veröffentlicht.

Mit dieser Kasuistik möchten wir dafür sensibilisieren, dass die Möglichkeit einer retinalen Venenthrombose als frühe hyperinflammatorische Reaktion einer COVID-19-Erkrankung in Betracht gezogen werden sollte. Auch bestätigt unser Fall die aktuelle Empfehlung der Thrombose- und Embolieprophylaxe bei schwerer COVID-19-Infektion [10].

## Fazit für die Praxis

- Die aktuelle Studienlage zu retinalen Manifestationen bei COVID-19 ist sehr begrenzt. Bei vaskulären Pathologien des Fundus sollte auch an eine mögliche COVID-19-Erkrankung gedacht werden.
- SARS-CoV-2-RNA lässt sich in Tränenflüssigkeit nachweisen. Dies sollte als mögliche Infektionsquelle bedacht werden.
- Ophthalmologische Manifestationen könnten Frühsymptome einer schweren COVID‑19-Pneumonie sein.
